# Development of Antimicrobial Coatings from *Ficus carica* Latex for Improving the Quality of Dried Figs

**DOI:** 10.3390/foods14091562

**Published:** 2025-04-29

**Authors:** Yesuneh Gizaw, Rocío Casquete, María del Carmen Caballero, María de Guía Córdoba, María José Benito

**Affiliations:** 1 Nutrición y Bromatología, Escuela de Ingenierías Agrarias, Universidad de Extremadura, Avd. Adolfo Suárez s/n, 06007 Badajoz, Spain; ygizawch@alumnos.unex.es (Y.G.); mcaballerod@unex.es (M.d.C.C.); mdeguia@unex.es (M.d.G.C.); mjbenito@unex.es (M.J.B.); 2 Instituto Universitario de Investigación en Recursos Agrarios (INURA), Universidad de Extremadura, Avd. de la Investigación, 06006 Badajoz, Spain

**Keywords:** *Ficus carica*, latex, coatings, antimicrobial, dried figs

## Abstract

To carry out this work, latex from *Ficus carica* was obtained for the production of coatings, the characteristics of the produced coatings were analyzed, and their application on dried figs was evaluated. Work was conducted to obtain latex and produce coatings, optimizing the mixture and determining its properties. Additionally, the shelf life of coated fruits was studied. The results showed that the coatings had a milky white color, a thickness between 0.04 mm and 0.09 mm, a moisture content close to 25%, and a water solubility ranging from 80% to 98.73%. The 10% latex coatings showed better elasticity and resistance, being selected for the shelf life study. The optimal formulations selected were 28, 29, and 31, all with 10% latex. These coatings exhibited interesting antimicrobial activities against bacteria *Escherichia coli*, *Staphylococcus aureus*, and *Salmonella choleraesuis* and antifungal activity against *Botrytis cinerea*, *Penicillium expansum*, *Aspergillus flavus*, and *Monilinia fructicola*. When applied to dried figs, it was observed that the appearance of the figs did not visibly change. Antioxidant activity was highest in batch 28, which also showed less microbiology and lower hardness at 60 days. Thus, coatings not only helped maintain the natural color of the fruits but also preserved their freshness and overall quality for a longer period. This makes them an effective and sustainable solution for the food industry.

## 1. Introduction

*Ficus carica*, commonly known as the fig, is the most commercially significant species within the genus *Ficus*, one of the largest genera of angiosperms, due to its unique fruit structure—closed inflorescences with a succulent, hollow receptacle [1]. *F. carica* is marketed both fresh and dried, consumed directly or as part of various culinary dishes [2,3]. Turkey is the largest global producer of figs, followed by Egypt, Morocco, Iran, Algeria, and Spain. In Europe, Spain leads fig production, followed by Greece and Italy, and global consumption of this fruit has increased over the past decade [4]. The fig processing industry generates numerous byproducts such as leaves, pulp, shells, seeds, and latex, which can be utilized for various applications [5]. These biowastes can be extracted and used as value-added ingredients [6], enhancing the economic performance of production and promoting a circular economy in line with current consumer preferences for sustainability.

Latex, a milky juice exuded from various parts of the plant, including its fruits, stems, and seeds, contains diverse secondary metabolites such as terpenoids, organic acids, alkaloids, phytosterols, fatty acids, tannins, sterols, enzymes, and amino acids [7,8,9]. This juice is secreted in appreciable quantities, primarily serving to protect and self-heal the plant against external aggressions. The various metabolites present in latex are responsible for its biological properties, including antioxidant, cytotoxic, antiviral, antimicrobial, antiparasitic, antidiabetic, and wound-healing effects [7,8,10,11]. In addition, latex has been shown to have selective cytotoxic effects on cancer cells, especially breast, prostate, and colon cancer cells, without affecting normal cells [10,12].

Therefore, *Ficus carica* latex is an important matrix to study for developing materials for widespread use in various food applications, such as food coatings. Recent literature includes several studies on the use of natural antioxidants extracted from agro-food byproducts for active food packaging applications based on biodegradable materials [13]. The use of biomaterials is a sustainable development approach that aids in environmental preservation due to their advantages, including wide availability, non-toxicity, biodegradability, and biocompatibility [14].

Edible coatings are primarily used to enhance the appearance of fruits and extend their shelf life, as the perishable nature of fruits results in post-harvest losses estimated at 44% [15,16]. These coatings could delay ripening-related changes and microbial contamination. Currently, due to the non-degradability of synthetic packaging materials, interest in natural resources for manufacturing biodegradable edible coatings has increased [17,18]. Research should focus on developing edible coatings with high mechanical strength, gas barrier properties, lightness, smoothness, water resistance, and transparency [19,20,21]. These are increasingly recognized as a sustainable and cost-effective alternative to conventional plastic packaging, particularly in the preservation of fresh and minimally processed fruits and vegetables [20,22]. Their production typically involves simple and low-cost techniques such as casting or soaking and drying, which are easily adaptable to various scales of operation. These coatings are primarily formulated using bio-based materials—proteins, polysaccharides, and lipids—often sourced from readily available agro-industrial by-products like fruit peels and seeds, thus aligning with circular economy principles. Each material class contributes distinct functional properties: proteins provide resistance to lipid oxidation, polysaccharides are effective gas barriers though often brittle, and lipids offer excellent moisture resistance but limited gas barrier efficiency [20,22]. Combining these biopolymers in the form of complexes improves not only barrier functions but also the mechanical strength and thermal stability of the coatings, enhancing their performance under storage conditions [20]. While the base materials are widely accessible, the supply of functional additives such as essential oils can be variable, and the integration of non-thermal technologies like UV or γ-irradiation—though effective—may raise processing costs, especially for small-scale applications [22]. Nonetheless, the overall feasibility, biodegradability, and preservation effectiveness of edible coatings make them a promising strategy for food protection in both economic and environmental terms.

In the reviewed literature, no studies have been found on the use of edible coatings based on *F. carica* latex, only a few references using rubber or aloe vera latex. Adibi et al. [23] employed alpha-1,3-glucan polysaccharide derived from an enzymatic polymerization process as a functional additive for coating films based on natural rubber latex.

In this study, different coating mixtures based on latex obtained from *F. carica* were prepared, analyzing their antioxidant and antimicrobial characteristics, to be subsequently applied to the surface of dried figs, studying their preservation efficacy compared to control samples.

## 2. Materials and Methods

### 2.1. Plant Material

Spring pruning residues from fig trees (*Ficus carica*) originating from an industry plot located in the municipality of Don Benito, Badajoz, were used for latex extraction. The fruit selected for the shelf-life study were dried figs of the Calabacita from the same industry.

### 2.2. Bacterial and Fungal Strains

For this study, eight strains from the Spanish Type Culture Collection (*Staphylococcus aureus* CECT 976, *Bacillus cereus* CECT 131, *Listeria monocytogenes* CECT 911, *Listeria innocua* CECT 910, *Escherichia coli* CECT 4267, *Salmonella choleraesuis* CECT 4395, *Botrytis cinérea* CECT 20518, and *Penicillium expansum* CECT 2278) and three from the collection of the CAMIALI group from the University of Extremadura, Spain (*Aspergillus flavus* HG 144 M, *Monilinia fructicola* 362, and *Aspergillus niger* HG 185 M) were used.

### 2.3. Extraction of Latex from Fig Tree Pruning Byproducts

The fig tree can undergo two types of pruning: one in winter (formation and fruiting pruning) and another in spring (green pruning to remove excess vegetation). It was observed that direct latex collection was more effective during the spring. Therefore, the milky liquid expelled by the plant during pruning cuts was collected. For this purpose, latex was manually collected in 50 mL tubes from the cuts made during pruning.

### 2.4. Development of Protective Coatings Based on Latex

The parameters to consider were the amount of latex (minimum 1% and maximum 10%), the amount of citric acid (Panreac, Barcelona, Spain) (minimum 2% and maximum 3%), and the amount of glycerol (with a purity of 99%, Sigma-Alderich Chemie, Schnelldorf, Germany) (minimum 0% and maximum 1%) added to the formulation. Table 1 shows the different formulations tested.

Firstly, the collected latex was stabilized with a solution of creaming agents to prevent the paste from completely solidifying and to help maintain a smooth and malleable consistency free of impurities. To obtain these creaming agents, 3 g of polyethylene glycol 8000 (Scharlab SL, Sentmenat, Spain), 3 g of polyvinyl alcohol 88% (Termo Scientific, Omaha, NE, USA), 3 g of agar (Condolab, Madrid, Spain), 3 g of pectin (Sigma-Alderich), and 3 g of carboxymethyl cellulose (DS = 0.7, Termo Scientific) were added to a beaker, along with 100 mL of distilled water [24]. Subsequently, the flask was placed in a bath and heated for 10 min at 90–95 °C to homogenize the mixture. After 10 min, the mixture was diluted and the required percentage of latex (1%, 5%, or 10%) was added, followed by an additional 5 min in the bath to homogenize the mixture.

From this suspension, different combinations were made with citric acid (for its antioxidant, acidifying, and preservative functions) and glycerol (for its plasticizing function) to determine the optimal formulations for coating production. The concentrations of citric acid were 2%, 2.5%, and 3%, and the concentrations of glycerol were 0%, 0.5%, and 1% (Table 1). After preparing the different mixtures, they were heated to 80 °C for ten minutes, and 25 mL of the mixture was poured into 120 × 120 mm plates to form biopolymer films.

### 2.5. Study of the Suitability of Coatings

To study the suitability of the coatings, their physicochemical properties, such as color, thickness, moisture, and water solubility, were evaluated. The parameters studied included color using a DigiEye colorimeter from Verivide with DigiPix software 3.1 and CIELAB color space coordinates of the different formulations produced. Thickness was determined using a Sealey digital caliper (0–25 mm) with a precision of 0.001 mm. Moisture content was calculated by gravimetry after dehydration in an oven at 105 °C for 24 h, and water solubility was determined as the percentage of dry matter of the film soluble in water according to Hafsa et al. [25].

### 2.6. Antimicrobial Activity

To carry out the antibacterial activity, the culture medium for bacterial growth, Brain Heart Infusion (BHI) broth (Condalab, Madrid, Spain), was first prepared. Bacteria were grown at 37 °C for 24 h. The colony of each microorganism was then transferred to a peptone water solution until a concentration of 10^5^ CFU/mL was reached. Next, 1 mL of each suspension was pipetted into separate sterile Petri dishes to which 20 mL of molten BHI containing 1.5% agar (45 °C) was added. Circular movements were performed to ensure uniform distribution of bacteria across the plate. When the medium solidified, 10 μL of the different prepared latex combinations (Table 1), the mixture before being heated to 80°, were added. Citric acid and glycerol at the concentrations used in the combinations were used as negative controls. Finally, the plates were incubated at 37 °C for 24 h, and the diameter (mm) of the inhibition zone was measured.

To perform the antifungal activity, the molds were first grown on PDA (Potato Dextrose Agar) culture medium (Scharlab, Barcelona, Spain). For the inoculation of each mold, solutions of 1.5 mL of 10^6^ spores/mL were prepared from the molds grown on PDA plates. Each PDA plate was inoculated with 100 μL of the studied combinations and spread with a glass rod. The samples were allowed to dry completely on the plate for 30 min, and then 5 μL of the spore suspension of each mold was added. The inoculation of the spore suspension of each mold was used as a positive control, and citric acid at the concentrations used in the samples was used as negative controls. The molds were incubated in an oven at 25 °C for 7 days, and their diameter was measured horizontally and vertically over a period of 7 days. The results were expressed in mm of growth.

### 2.7. Shelf-Life Study of Dried Figs Coated with Selected Combinations

After carrying out several determinations and analyzing the properties of each of the prepared biopolymer films, the shelf-life study was carried out with the formulations that showed the best properties. For this purpose, 20 dried figs were used for each formulation, together with a control batch. The control batch was treated with sterile water. Each formulation was applied to the fruit by spraying. After application, the fruits were placed in trays of 10 fruits each and stored at room temperature for 60 days. During the storage period, samples were taken at 0, 30, and 60 days for microbiological analyses (mesophilic aerobic bacteria in PCA plates and yeasts and molds in PDA plates) and physicochemical analyses.

Color and moisture were determined as explained above. Texture, defined as force (N), was determined using a Stable Micro System texturometer model TA.XT. plus. For this, 3 measurements were taken from each whole fig sample using a 6 mm probe with a measurement depth of 10 mm, which was performed using a Stable Micro System texturometer model TA.XT. plus. To determine the antioxidant activity, an extraction of the compounds was carried out. For this, 10 g of dried fig were weighed, and 50 mL of 80% MetOH were added. Subsequently, the mixture was homogenized using an OmniMixer blender. The mixture was then subjected to ultrasound for 1 h in darkness. After this period, the mixture was filtered into a 50 mL Falcon tube, and the final volume was recorded. The antioxidant activity of the extracts diluted to 10 mg extract/mL with ethanol was determined by bleaching of the purple-colored solution of 1,1-diphenyl-2-picrylhydrazyl radical (DPPH) according to the method of [26]. Quantification was performed by plotting the values against a Trolox calibration curve. Results were expressed in mg of Trolox/100 g of extract. All experiments were conducted in triplicate. The titratable acidity of the figs was determined using an automatic titrator model 855 Robotic Titrosampler from Metrohm with Tiamo 2.5 software, connected to an 800 Dosino dispenser of 0.1 N sodium hydroxide until neutrality was reached. The preparation consisted of 2.5 g of chopped fig and 25 mL of distilled water (1:10). The result was expressed as g of malic acid/l and g of citric acid/l, as these are the predominant acids in the fruit. The pH of the fig was determined using the automatic titrator simultaneously with the titratable acidity. To measure the total soluble solids, a refractometer was used to measure the refraction of a sample, referencing the amount of soluble solids present in the sample, with results expressed in degrees Brix. Determination of reducing sugars was performed by weighing 1 g of fig and adding 10 mL of Milli-Q water in a 15 mL Falcon tube, which was left for 30 min. Subsequently, the mixture was centrifuged for 5 min at 4500 rpm. The supernatant was filtered into a new 15 mL Falcon tube. A 1:10 dilution was prepared in a 1.5 mL Eppendorf tube (containing 100 µL of sample and 900 µL of HPLC-grade H_2_O). The mixture was then filtered with a 0.45 µm nylon filter into an HPLC vial. Finally, the analysis was performed using HPLC [27].

### 2.8. Statistical Analysis

Statistical analysis of the data was carried out using SPSS for Windows, version 21.0 (IBM Corp., Armonk, NY, USA). Descriptive statistics of the data were determined, and the differences within and between groups were studied by one-way analysis of variance (ANOVA) and separated by Tukey’s honestly significant differences test (*p* ≤ 0.05). Principal component analysis (PCA) was performed on the correlation matrix of the variables rather than the covariance matrix, which inherently standardizes the data.

## 3. Results and Discussion

### 3.1. Development of Protective Coatings Based on Latex

The latex liquid expelled by the plant during spring pruning cuts was collected. For this purpose, latex was manually collected from the cuts made during pruning. The biopolymer films prepared with the different blends (Table 1) were observed and analyzed once formed on 120 × 120 mm plates.

The CIELAB color space coordinates of the different formulations were obtained, including lightness, the red–green coordinate, and the yellow–blue coordinate. The analysis was performed directly on the biopolymer films placed on 120 × 120 mm plates. Lightness values obtained ranged between 86 and 87, without significant differences in the different formulations. Regarding a coordinate (red–green coordinate), all values were below 1, with the minimum value being 0.20 (biopolymer film 20) and the maximum value being 0.74 (biopolymer films 41 and 43). Finally, for the b coordinate (yellow–blue coordinate), values ranged between 1.38 and 2.37, with the lowest value in biopolymer film 43 and the highest values in formulations 35 and 36, with no significant differences in any of these biopolymer films. Thus, these parameters did not seem very decisive for selecting a mixture.

The results for physical property analysis, such as thickness, moisture, and solubility of *Ficus carica* L. latex-based coatings, are shown in Table 2.

The thickness of the biopolymer films varies between 0.04 and 0.09 mm. The lowest value is observed in formulation 10/3/0 (0.04 ± 0.01), while several formulations exhibit the highest (*p* < 0.05) thickness of 0.09 mm, including 5/2/0.5, 5/2/1, 5/2.5/1, 10/2/0.5, and 1/2/0.5, among others. The results indicate that the composition of the materials used influences the final structure of the biopolymer film [28]. Moisture values range between 21.44% (10/2/0.5) and 32.19% (1/3/0). Generally, biopolymer films with lower moisture content tend to be those with formulations containing 10 and 5% latex and 3% citric acid, while formulations with values above 30% include 5/2/1 (30.81 ± 0.76) and 1/3/0 (32.19 ± 6.59). The variability in moisture could be related to the water retention capacity of the components used and their influence on the film structure. Solubility values show greater dispersion, ranging between 77.07% (10/2/0) and 98.73% (5/3/1). It is observed that formulations with higher content of certain compounds tend to have greater solubility, which could be related to the interaction between the polymers and the biopolymer film matrix. Formulations with higher solubility may be more susceptible to degradation in humid environments, which could be a determining factor in their application [28].

Biopolymer films exhibit considerable variability in their physical properties, depending on the formulation used. Thickness is relatively stable in most samples, while moisture and solubility show significant differences. It is important to consider these characteristics in relation to the final use of the biopolymer films, as properties such as solubility can influence their stability and functionality in specific applications. However, blends with 1% latex and different percentages of citric acid and glycerol showed biopolymer films that were not consistent enough to release, or those that did release broke due to the weakness of the biopolymer film, and some of the 5% films were released but subsequently broke because they were too sticky and not sufficiently elastic. For this reason, the 1% and 5% latex blends were discarded.

**Figure 1:**
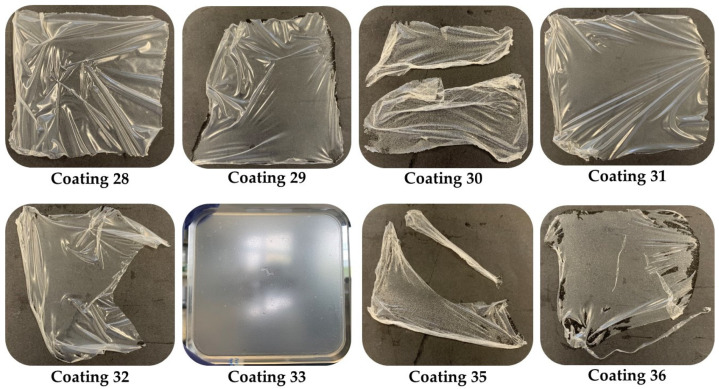
Plates with the 10% latex coatings.

Figure 1 shows the plates of the 10% latex films; all of them were released except for film 33, observing that this percentage gave more elasticity and resistance to the films. Some of these coatings were completely released, as shown below.

Based on the physicochemical properties observed, it was determined that only the formulations containing 10% latex (specifically mixtures 28 to 36) would be subjected to antimicrobial activity testing.

The antibacterial activity was tested against pathogenic bacteria from the Spanish Type Culture Collection, specifically, *Staphylococcus aureus* CECT 976, *Bacillus cereus* CECT 131, *Listeria monocytogenes* CECT 911, and *Listeria innocua* CECT 910 as Gram-positive bacteria, and *Escherichia coli* CECT 4267 and *Salmonella choleraesuis* CECT 4395 as Gram-negative bacteria. Regarding the results obtained, no antibacterial activity was observed in any of the samples tested against *Bacillus cereus*, *Listeria monocytogenes*, or *Listeria innocua*. However, activity was observed against *Salmonella choleraesuis* in all tested combinations and against *Escherichia coli* in combinations 31 and 33, which showed inhibition zones of 6 ± 0.10 mm and 4 ± 0.05 mm, respectively. For *Staphylococcus aureus*, only formulation 34, containing 3% citric acid, showed activity, with an inhibition zone of 3 ± 0.01 mm. This indicates that most formulations do not have a significant effect on these bacteria, which could be related to the composition of the biofilms and their mechanism of action. No activity was observed in the controls performed with citric acid alone and glycerol alone. The analyzed biofilms showed variability in their antibacterial activity depending on the target bacteria. There is limited information in the literature regarding the antibacterial activity of fig latex; however, there are studies involving latex from other *Ficus* species or rubber. Salem et al. [29] evaluated the activity of nanoparticles from aqueous extracts of latex and leaves of *Ficus sycomorus*. Their results align with ours, as they also observed higher activity against *Salmonella* sp. and *Escherichia coli* among the Gram-negative bacteria and *Staphylococcus aureus* among the Gram-positive bacteria. On the other hand, Ref. [30] conducted a comparative study of the antibacterial effects of latex from *Calotropis procera* and also corroborated that the antibacterial activity of latex was moderate.

Regarding antifungal activity, analyses were carried out to evaluate the activity against molds responsible for fruit spoilage and mycotoxigenic molds (Table 3 and Table 4). Table 3 presents the growth (in mm) of *Monilinia fructicola* and *Botrytis cinerea* after 7 days of incubation at 25 °C under various conditions, including a control, treatments with citric acid, and biofilms with different formulations. Overall, it is observed that some biofilms were able to reduce fungal growth compared to the control, although the effects vary depending on the species and the specific formulation.

**Figure 2:**
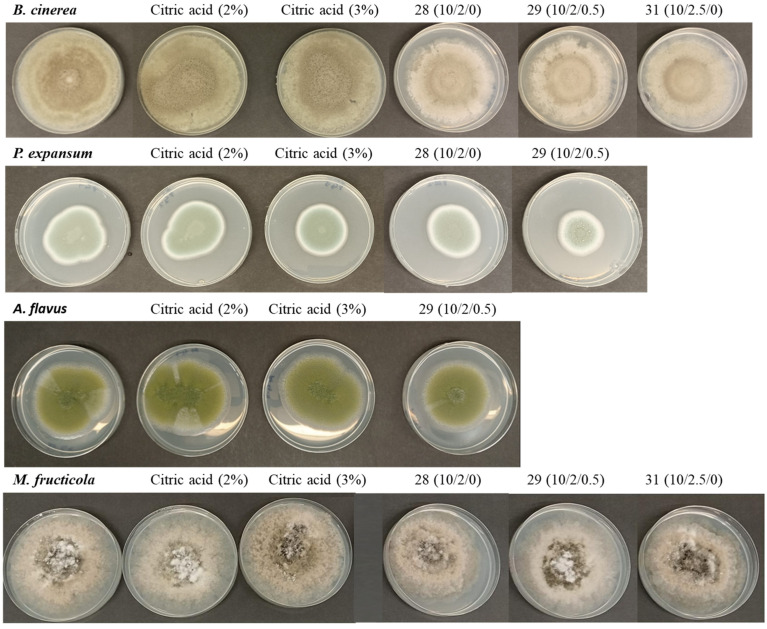
Growth of mold strains in PDA medium after 7 days of incubation. Controls and samples with the biofilm formulations that showed activity.

For *M. fructicola*, the growth in the control was 80.50 ± 2.09 mm, while citric acid at 2% and 3% showed a slight reduction (79.10 ± 1.47 mm and 78.00 ± 0.89 mm, respectively), with no statistically significant differences compared to the control. However, some biofilms were more effective in reducing growth, particularly formulation 28 with 75.33 ± 0.29 mm and 29 with 76.05 ± 0.18 mm, which showed significant differences (*p* < 0.05) compared to the control (Figure 2). In the case of *B. cinerea*, the control also showed a growth of 81.80 ± 1.86 mm, and citric acid at 2% and 3% did not have a significant inhibitory effect. However, some biofilms significantly reduced growth, especially formulations 28 and 29, which showed significant differences compared to the control (Figure 2). These results indicate that some biofilms were able to reduce the growth of *M. fructicola* and *B. cinerea*, but their effectiveness depends on the specific formulation. Formulations 28 and 29 were the most effective in inhibiting the growth of both fungi. This suggests the need to optimize the formulations to enhance their antifungal activity.

There are no studies on the inhibition of these fungi with latex, but [31] studied the antifungal activity of a proteolytic fraction P1G10 from the latex of *Vasconcellea cundinamarcensis* against the cell growth and cell wall integrity of *Botrytis cinerea,* and they propose it as a promising antifungal agent for controlling disease-causing agents in the food agroindustry.

Table 4 presents the growth (in mm) of *Aspergillus niger*, *Aspergillus flavus*, and *Penicillium expansum* after 7 days of incubation at 25 °C under various treatments, including a control, citric acid, and biofilms with different formulations. It is observed that the effects of the treatments vary depending on the fungal species, and some formulations significantly reduce growth compared to the control.

For *A. niger*, the growth in the controls and samples did not show a clear inhibitory effect, suggesting that these formulations were not effective on this species. However, for *A. flavus*, some biofilms were able to decrease its growth, particularly formulation 29, with 57.83 ± 0.58 mm, which showed significant differences (*p* < 0.05) compared to the control (Figure 2). Finally, for *P. expansum*, biofilm formulations 29 and 31 showed significant differences (*p* < 0.05) compared to the control (Figure 2). The results indicate that some biofilm formulations were able to reduce the growth of *A. flavus* and *P. expansum*, particularly formulation 29, which showed the greatest inhibition in both species. These findings highlight the importance of optimizing formulations and evaluating their impact on each fungal species to improve their effectiveness as antifungal agents. There are no studies on the effect of latex on these mycotoxigenic species; however, it is known that the bioactive compounds in plant latex are a potential source of antifungals against postharvest pathogens [32].

### 3.2. Shelf-Life Study of Dried Figs Coated with Selected Combinations

**Figure 3:**
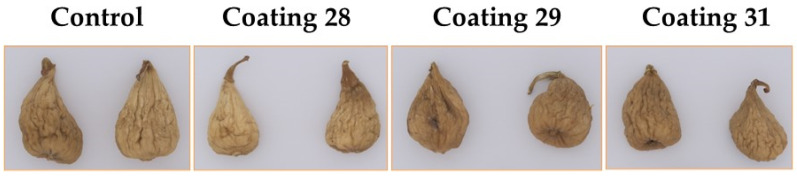
Samples of the dried figs after application of the different coatings.

After analyzing the obtained biofilms, the optimal formulations with 10% latex were chosen for the shelf-life study. The selected formulations were 28, 29, and 31. Regarding citric acid content, formulations 28 and 29 were constituted of 2% citric acid, while formulation 31 contained 2.5%. Additionally, glycerol was present only in formulation 29 at a minimal amount of 0.5%. Figure 3 shows the different batches of figs after applying the selected coatings by spraying. As observed, the application of the coatings did not alter the visual appearance of the dried figs. Biofilms are typically transparent or slightly opaque, allowing the natural appearance of the fruit to remain visible. This is important for consumer acceptance, as coated fruits retain their original color and shine [33].

Microbiological counts of mesophilic aerobic bacteria (Log CFU/g) during the shelf-life study of different batches of dried figs, evaluated at days 0, 30, and 60, are presented in Table 5. Uncoated control was compared with different coatings formulated with biofilms.

Regarding mesophilic aerobic bacteria, all batches had levels above 3 Log CFU/g on day 0 of coating application. After 30 days, these values significantly decreased in the batch with coating 28, which showed significantly lower values (*p* < 0.05) compared to the other batches. On day 60, a significant reduction in mesophilic aerobic bacteria was observed, with the batch coated with formulation 28 being the only one where bacterial levels were below the detection limit.

Regarding mold counts, on day 0, all batches had values close to 3 Log CFU/g, with the control and batch 28 showing the highest values (*p* < 0.05). These levels significantly decreased over the storage period, reaching values greater than 1 Log CFU/g in the control batch and batch 31 on day 60. Coating 28 showed the greatest effect against mold growth, as it was the only batch where no mold growth was detected at the end of the storage period. Yeasts were below the detection limit in all analyzed batches.

Formulate coatings with natural antimicrobial agents that help prevent the growth of pathogens and molds on the surface of the fruit [34]. These antimicrobial agents can include various compounds such as essential oils, plant extracts, and other biopolymers with antimicrobial properties.

These results suggest that the evaluated coatings are effective and represent an additional benefit in terms of the microbiological safety and stability of dry figs during storage.

The analysis of color parameters during the shelf-life study shows differences in the L (lightness), a* (red–green), and b* (yellow–blue) coordinates, indicating changes in the appearance of the figs throughout the storage period (Table 6).

The lightness (L) decreased over time in all batches, suggesting a progressive darkening of the fruit. In the control batch, lightness decreased from 57.67 on day 0 to 46.02 on day 60, reflecting evident deterioration. However, the coated batches showed a smaller decrease in lightness, especially coating 29, which maintained more stable values (51.15 on day 0 and 51.42 on day 60). This suggests that the coatings may help delay the darkening of the fruit, which is a positive indicator in terms of visual quality. Regarding the a* coordinate, which indicates the intensity of the red color, a progressive increase was observed in all batches throughout storage. In the control batch, values increased from 13.56 on day 0 to 16.22 on day 60, indicating a shift towards more reddish tones. The coated batches also showed an increase in this coordinate, but without significant differences between them. The b* coordinate, representing the intensity of the yellow color, showed a slight decrease, not significant, over time in most batches, which may be related to the degradation of natural fruit pigments.

The results suggest that the applied coatings helped reduce the loss of lightness and maintain more stable color characteristics of the figs during storage compared to the control batch. Coatings play a crucial role in protecting the color of fruits during storage and distribution [33]. Coatings act as a physical barrier that limits the fruit’s exposure to oxygen, which is essential for preventing the oxidation of natural pigments. This not only enhances visual appearance but can also increase consumer acceptance and reduce food waste.

Table 7 presents the evaluation of moisture, hardness, and antioxidant activity of dehydrated figs during 60 days of storage, revealing significant differences among the analyzed batches. The applied coatings appear to influence moisture retention, texture, and preservation of antioxidant compounds over time.

Initial moisture varied among the batches, with the control presenting 24.31%, while the coatings showed higher values, especially batch 29 with 30.85%. However, as storage progressed, all batches showed a reduction in moisture content, although the coated batches tended to retain more water than the control. At the end of the 60 days, the control had 18.53%, while batch 29 maintained a higher level (19.08%). This suggests that the coatings may help reduce water loss of the product over time.

In terms of hardness, initial values were relatively low in all batches but increased significantly after 60 days, especially in the control batch (1.12 N), indicating a progressive hardening of the fruit. The coatings showed lower hardness values at the end of storage, with batch 28 reaching 0.57 N and batch 29 reaching 0.70 N. This suggests that the coatings may have reduced the rate of hardening, possibly due to greater moisture retention and less dehydration.

The behavior of antioxidant activity varied among the batches. In the control, antioxidant activity decreased drastically from 27.35 mg Trolox/100 g at the beginning to only 4.23 mg Trolox/100 g at the end of storage, indicating significant degradation of antioxidant compounds over time. In contrast, the coated batches showed better preservation of antioxidant activity, with batch 28 reaching a value even higher than the initial after 60 days (34.03 mg Trolox/100 g), while batches 29 and 31 also maintained high levels (27.31 and 27.58 mg Trolox/100 g, respectively). These results indicate that the coatings may have protected the antioxidant compounds, possibly by reducing exposure to oxidation or degradation caused by moisture loss.

The results suggest that the coatings applied to dried figs have a positive impact on moisture conservation, reduction in hardening, and preservation of antioxidant activity. In particular, batch 29 showed the best balance between water retention, lower hardness, and stable antioxidant activity. These findings highlight the importance of using coatings to improve the quality and shelf life of dried figs.

By reducing moisture loss, coatings help maintain the texture and color of the fruit. Dehydration can cause the fruit to become dull and lose its natural shine, but coatings preserve internal moisture, thus maintaining its fresh appearance. In addition, coatings can contain natural antioxidants, which help protect the fruit’s pigments against oxidative damage [33].

The study of the evolution of pH and citric and malic acids in dehydrated figs during storage reveals significant variations among different batches and over time (Table 8). These parameters are crucial as they influence microbiological stability, sensory characteristics, and the quality of the final product.

The pH remained stable until day 30, but a significant decrease was observed in all batches by day 60. The citric acid content varied throughout storage. Initially, the coated batches had slightly higher concentrations compared to the control (1.03–1.14 g/L vs. 0.76 g/L). By day 30, all batches experienced an increase in citric acid concentration, but on day 60, citric acid levels decreased in all batches. The behavior of malic acid was similar to that of citric acid. The results indicate that the applied coatings can slightly modify the pH evolution since a tendency was observed in coatings 28 and 29 to delay this process.

The evolution of soluble solids (°Brix), glucose, and fructose in dehydrated figs over 60 days of storage shows significant variations that may be related to changes in sugar concentration and water migration within the fruit (Table 9).

Soluble solids values increased in all batches, reaching between 85.37 and 89.03 °Brix by the end of the storage period. This suggests a progressive concentration of sugars due to moisture loss. The higher initial values in the control could be due to the absence of a protective barrier that retained water content. Glucose showed a drastic decrease in all batches after day 0. However, by day 30, values dropped significantly and this trend continued until day 60. This decrease may be related to sugar degradation processes during storage or the conversion of glucose into other compounds. The coatings do not seem to prevent this reduction, although they may modulate the rate of degradation.

The behavior of fructose was similar to that of glucose. This behavior supports the hypothesis that reducing sugars are undergoing some form of transformation or degradation during storage, possibly due to non-enzymatic browning reactions, such as the Maillard reaction, or interactions with other compounds present in the dehydrated fig matrix.

The coatings seem to affect the initial concentrations of soluble solids and sugars but do not prevent their evolution during storage. This suggests that while the coatings may influence product preservation, they are not completely effective in halting sugar transformation over the storage period.

### 3.3. Multivariate Analysis of the Parameters Studied in Coated Dried Figs

Principal component analysis was carried out for all the variables analyzed in the shelf-life analysis at 60 days of storage.

**Figure 4:**
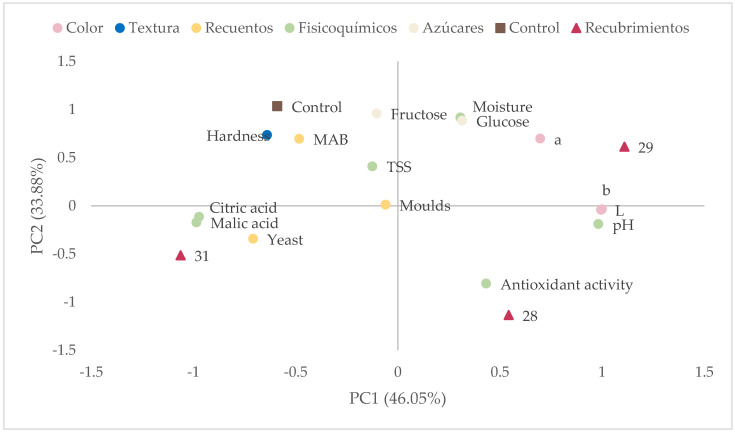
Plane defined by principal components 1 and 2 (PC1 and PC2) showing the parameters analyzed and the batches studied after 60 days (control, coating 28, coating 29, and coating 31). MAB: mesophilic aerobic bacteria count; TSS: total soluble solids.

Figure 4 shows principal component 1 (PC 1), which explains 46.05% of the variance in the analysis, is positively correlated with color and pH values, associating with the fig batch coated with formulation 29. On the contrary, the acidity parameters and mold counts located on the axis of this component are associated with the dried fig samples coated with formulation 31.

Principal component 2 (33.88% of the variance) is related to the values of sugar content, moisture, hardness, and mesophilic aerobic bacteria (MAB) associated with the control batch (Figure 4). Antioxidant activity is on the opposite plane, correlated with the batch coated with formulation 28. This batch proved to be the most effective because, in addition to having higher antioxidant activity, it presented lower microbiological counts and reduced hardness at 60 days.

## 4. Conclusions

The results obtained in the present study are of great importance for sustainable solutions in the food industry, which include using natural novel coatings based on *F. carica* latex that help maintain the natural color of the fruits but also preserve their freshness and overall quality for a longer period of time.

The optimization of the formulations allowed selecting coatings with 10% of latex, and formulations 28, 29, and 31 were the coatings with better elasticity and resistance for use in the shelf-life study of fruit. Additionally, they improved quality and safety by exhibiting antimicrobial activities and maintaining the lightness, color, hardness, and antioxidant activity of the figs during 60 days of storage, highlighting coating 28.

Consequently, the results obtained are very promising, but future studies are needed to determine whether the coatings are completely safe for food use, as well as to provide additional physicochemical analyses such as water vapor transmission rate (WVTR), optical transparency, and the mechanical and thermal stability of the tested coatings.

## Figures and Tables

**Table 1:** Formulations tested for obtaining coatings with 1%, 5%, and 10% latex.

Test Number	Volume in Plate (mL)	Latex	Citric Acid	Glycerol
37	25	1%	2%	0%
38	25	1%	2%	0.5%
39	25	1%	2%	1%
40	25	1%	2.5%	0%
41	25	1%	2.5%	0.5%
42	25	1%	2.5%	1%
43	25	1%	3%	0%
44	25	1%	3%	0.5%
45	25	1%	3%	1%
19	25	5%	2%	0%
20	25	5%	2%	0.5%
21	25	5%	2%	1%
22	25	5%	2.5%	0%
23	25	5%	2.5%	0.5%
24	25	5%	2.5%	1%
25	25	5%	3%	0%
26	25	5%	3%	0.5%
27	25	5%	3%	1%
28	25	10%	2%	0%
29	25	10%	2%	0.5%
30	25	10%	2%	1%
31	25	10%	2.5%	0%
32	25	10%	2.5%	0.5%
33	25	10%	2.5%	1%
34	25	10%	3%	0%
35	25	10%	3%	0.5%
36	25	10%	3%	1%

**Table 2:** Results of physical property determination (thickness (mm), moisture (%), and solubility (%)) of *Ficus carica* L. latex-based coatings.

	Thickness			Moisture			Solubility		
Coating	Mean		SD *	Mean		SD	Mean		SD
19 (5/2/0)	0.06	±	0.01 ^a–c^	30.55	±	1.72	83.75	±	1.19 ^d,e^
20 (5/2/0.5)	0.09	±	0.01 ^a^	23.48	±	1.00	85.18	±	1.27 ^c–e^
21 (5/2/1)	0.09	±	0.01 ^a,b^	30.81	±	0.76	88.01	±	1.07 ^b–d^
22 (5/2.5/0)	0.06	±	0.01 ^a–c^	23.50	±	6.92	86.59	±	1.68 ^c–e^
23 (5/2.5/0.5)	0.08	±	0.01 ^a,b^	22.66	±	0.25	88.32	±	1.41 ^b–d^
24 (5/2.5/1)	0.09	±	0.02 ^a,b^	29.51	±	2.07	89.86	±	1.84 ^a–d^
25 (5/3/0)	0.08	±	0.01 ^a,b^	30.59	±	7.21	91.46	±	0.06 ^a–d^
26 (5/3/0.5)	0.08	±	0.02 ^a–c^	22.63	±	0.11	96.93	±	0.15 ^a,b^
27 (5/3/1)	0.08	±	0.00 ^a,b^	25.93	±	0.45	98.73	±	0.73 ^a^
28 (10/2/0)	0.08	±	0.01 ^a–c^	28.69	±	5.39	77.07	±	0.47 ^e^
29 (10/2/0.5)	0.09	±	0.01 ^a,b^	21.44	±	0.72	88.18	±	0.03 ^b–d^
30 (10/2/1)	0.09	±	0.00 ^a,b^	26.41	±	1.39	91.23	±	1.90 ^a–d^
31 (10/2.5/0)	0.07	±	0.01 ^a–c^	24.49	±	1.78	82.88	±	0.74 ^d,e^
32 (10/2.5/0.5)	0.08	±	0.01 ^a,b^	21.96	±	0.21	89.03	±	0.06 ^a–d^
33 (10/2.5/1)	0.09	±	0.01 ^a,b^	25.74	±	1.44	92.55	±	0.90 ^a–d^
34 (10/3/0)	0.04	±	0.01 ^c^	28.38	±	1.72	85.16	±	0.36 ^c–e^
35 (10/3/0.5)	0.09	±	0.01 ^a^	21.73	±	0.45	91.70	±	0.38 ^a–d^
36 (10/3/1)	0.09	±	0.00 ^a,b^	22.67	±	0.22	90.41	±	3.08 ^a–d^
37 (1/2/0)	0.05	±	0.01 ^b,c^	23.08	±	1.52	83.70	±	0.24 ^d,e^
38 (1/2/0.5)	0.09	±	0.01 ^a,b^	27.37	±	1.66	93.85	±	7.14 ^a–c^
39 (1/2/1)	0.07	±	0.01 ^a–c^	27.93	±	1.92	93.70	±	6.00 ^a–c^
40 (1/2.5/0)	0.06	±	0.01 ^a–c^	26.70	±	0.49	86.78	±	1.16 ^c–e^
41 (1/2.5/0.5)	0.07	±	0.01 ^a–c^	22.51	±	0.13	94.77	±	5.96 ^a–c^
42 (1/2.5/1)	0.07	±	0.02 ^a-c^	23.76	±	0.76	89.94	±	1.00 ^a–d^
43 (1/3/0)	0.07	±	0.02 ^a-c^	32.19	±	6.59	88.99	±	0.76 ^a–d^
44 (1/3/0.5)	0.09	±	0.01 ^a,b^	22.99	±	0.54	91.88	±	0.30 ^a–d^
45 (1/3/1)	0.08	±	0.02 ^a,b^	24.01	±	1.13	98.65	±	0.94 ^a^

* SD: standard deviation; superscripts ^a,b,c,d,e^: significant differences (*p* < 0.05) by Tukey’s test among coatings. Sample codes indicate formulations by compositional percentages of the three main compounds (% latex/% citric acid/% glycerol).

**Table 3:** Growth (mm) of *Monilinia fructicola* 362 and *Botrytis cinerea* CECT 20518 after 7 days of incubation at 25 °C.

	*M. fruticola*			*B. cinerea*		
Samples ^a^	Mean		SD *	Mean		SD
Control	80.50	±	2.09 ^a^	81.80	±	1.86 ^a^
Citric acid (2%)	79.10	±	1.47 ^ab^	79.90	±	2.01 ^a^
Citric acid (3%)	78.00	±	0.89 ^ab^	79.00	±	0.77 ^ab^
It is corrects28 (10/2/0)	75.33	±	0.29 ^b^	69.00	±	2.69 ^b^
29 (10/2/0.5)	76.05	±	0.18 ^b^	73.50	±	1.32 ^b^
30 (10/2/1)	78.50	±	1.32 ^ab^	81.50	±	2.29 ^a^
31 (10/2.5/0)	76.00	±	0.87 ^b^	76.17	±	1.97 ^ab^
32 (10/2.5/0.5)	76.33	±	1.04 ^ab^	83.33	±	0.29 ^a^
33 (10/2.5/1)	78.83	±	1.26 ^ab^	82.33	±	0.58 ^a^
34 (10/3/0)	77.17	±	0.76 ^ab^	80.83	±	1.61 ^a^
35 (10/3/0.5)	77.17	±	2.57 ^ab^	78.33	±	0.83 ^ab^
36 (10/3/1)	77.83	±	1.04 ^ab^	78.83	±	0.92 ^ab^

* SD: standard deviation. ^a,b^ Mean values with different superscripts are significantly different (*p* < 0.05) between samples within the same day of incubation at 25 °C. a% latex/% citric acid/% glycerol.

**Table 4:** Growth (mm) of *Aspergillus niger* HG 185 M, *Aspergillus flavus* HG 144 M, and *Penicillium expansum* CECT 2278 after 7 days of incubation at 25 °C.

	*A. niger*			*A. flavus*			*P. expansum*		
Samples ^a^	Mean		SD *	Mean		SD	Mean		SD
Control	74.40	±	1.08	67.10	±	4.08 ^a^	50.50	±	5.68 ^a^
Citric acid (2%)	78.40	±	0.42	65.10	±	6.40 ^a^	47.83	±	4.51 ^a^
Citric acid (3%)	71.30	±	1.99	63.80	±	3.52 ^ab^	45.40	±	1.74 ^ab^
28 (10/2/0)	78.33	±	2.89	62.00	±	3.28 ^ab^	43.17	±	1.43 ^b^
29 (10/2/0.5)	78.50	±	2.18	57.83	±	0.58 ^b^	40.00	±	5.67 ^c^
30 (10/2/1)	79.50	±	1.32	60.17	±	0.29 ^ab^	45.17	±	2.17 ^ab^
31 (10/2.5/0)	80.00	±	0.87	60.83	±	1.44 ^ab^	42.17	±	3.75 ^bc^
32 (10/2.5/0.5)	80.17	±	1.44	59.50	±	2.65 ^ab^	43.17	±	1.43 ^bc^
33 (10/2.5/1)	79.50	±	1.50	60.00	±	4.00 ^ab^	44.90	±	0.84 ^bc^
34 (10/3/0)	79.17	±	2.75	64.17	±	3.82 ^ab^	46.17	±	5.48 ^ab^
35 (10/3/0.5)	79.17	±	2.75	63.33	±	3.82 ^ab^	44.67	±	1.15 ^bc^
36 (10/3/1)	77.33	±	1.04	65.00	±	2.50 ^a^	44.33	±	2.36 ^bc^

* SD: standard deviation. ^a,b,c^ Mean values with different superscripts are significantly different (*p* < 0.05) between samples within the same day of incubation at 25 °C. a% latex/% citric acid/% glycerol.

**Table 5:** Microbiological counts expressed in Log CFU/g from the shelf-life study of the batches of dried figs analyzed.

Batches	Mesophilic AEROBIC Bacteria								
	Day 0			Day 30			Day 60		
	Mean		SD *	Mean		SD	Mean		SD
Control	3.30	±	0.03 ^a^	3.68	±	0.03 ^a^	2.39	±	0.12 ^b^_1_
Coating 28 (10/2/0)	3.36	±	0.03 ^a^	3.21	±	0.09 ^a^	0.00	±	0.00 ^b^_3_
Coating 29 (10/2/0.5)	3.28	±	0.00 ^a^	3.50	±	0.12 ^a^	2.15	±	0.21 ^b^_2_
Coating 31 (10/2.5/0)	3.19	±	0.06	3.36	±	0.15	2.54	±	0.09 _1_
	**Molds**								
	**Day 0**			**Day 30**			**Day 60**		
	Mean		SD *	Mean		SD	Mean		SD
Control	2.95	±	0.07 ^b^_1_	3.65	±	0.03 ^a^_1_	2.00	±	0.00 ^a^_2_
Coating 28 (10/2/0)	3.23	±	0.16 ^a^_1_	2.00	±	0.00 ^b^_4_	0.00	±	0.00 ^c^_2_
Coating 29 (10/2/0.5)	2.69	±	0.12 ^b^_2_	3.31	±	0.05 ^a^_2_	1.00	±	0.00 ^c^_1_
Coating 31 (10/2.5/0)	2.54	±	0.09 ^a^_3_	2.80	±	0.14 ^a^_3_	1.15	±	0.21 ^b^_1_

* SD: standard deviation; ^a,b,c^ Values with different superscripts show significant differences (*p* < 0.05) between sampling days within the same batch. _1,2,3,4_ Values with different subscripts show significant differences (*p* < 0.05) according to the Tukey test between different batches on a sampling day.

**Table 6:** Results of the color coordinates of the shelf-life study of the batches of figs analyzed.

Batches	Lightness L								
	Day 0			Day 30			Day 60		
	Mean		SD *	Mean		SD	Mean		SD
Control	57.67	±	6.72 ^a^	58.13	±	6.41 ^a^	46.02	±	2.07 ^b^
Coating 28 (10/2/0)	57.77	±	5.38	59.08	±	6.65	49.56	±	2.09
Coating 29 (10/2/0.5)	51.15	±	5.93	51.19	±	1.18	51.42	±	3.67
Coating 31 (10/2.5/0)	54.11	±	3.12	52.18	±	1.35	45.35	±	4.01
	Red–green coordinate a *								
	**Day 0**			**Day 30**			**Day 60**		
	Mean		SD *	Mean		SD	Mean		SD *
Control	13.56	±	1.32 ^b^_2_	15.06	±	1.91 ^a^	16.22	±	1.22 ^a^
Coating 28 (10/2/0)	14.28	±	0.44 _1_	15.42	±	1.05	16.11	±	0.94
Coating 29 (10/2/0.5)	14.38	±	1.26 _1_	15.25	±	0.65	16.32	±	0.30
Coating 31 (10/2.5/0)	14.59	±	0.90 _1_	15.40	±	0.22	15.96	±	1.59
	Yellow–blue coordinate b *								
	**Day 0**			**Day 30**			**Day 60**		
	Mean		SD *	Mean		SD	Mean		SD *
Control	32.36	±	0.76	31.86	±	4.72	29.36	±	2.36
Coating 28 (10/2/0)	32.95	±	2.27	32.96	±	1.17	31.20	±	1.66
Coating 29 (10/2/0.5)	29.78	±	3.00	30.74	±	1.61	31.89	±	1.95
Coating 31 (10/2.5/0)	32.30	±	0.51	31.95	±	0.26	28.64	±	4.82

* SD: standard deviation; ^a,b^ Values with different superscripts show significant differences (*p* < 0.05) between sampling days within the same batch. _1,2_ Values with different subscripts show significant differences (*p* < 0.05) according to the Tukey test between different batches on a sampling day.

**Table 7:** Results of moisture (%), hardness (N), and antioxidant activity (mg Trolox/100 g) of dried figs during the 60 days of storage.

Batches	Moisture								
	Day 0			Day 30			Day 60		
	Mean		SD *	Mean		SD	Mean		SD *
Control	24.31	±	0.31 ^a^_3_	19.11	±	0.03 ^b^	18.53	±	0.81 ^b^
Coating 28 (10/2/0)	26.35	±	0.26 ^a^_2,3_	17.62	±	1.54 ^b^	16.95	±	1.56 ^b^
Coating 29 (10/2/0.5)	30.85	±	1.67 ^a^_1_	20.43	±	3.48 ^b^	19.08	±	1.29 ^b^
Coating 31 (10/2.5/0)	29.06	±	0.53 ^a^_1,2_	18.41	±	1.40 ^b^	17.45	±	0.50 ^b^
	**Hardness**								
	**Day 0**			**Day 30**			**Day 60**		
	Mean		SD *	Mean		SD	Mean		SD *
Control	0.40	±	0.15 ^b^_1,2_	0.27	±	0.09 ^b^_2_	1.12	±	0.13 ^a^
Coating 28 (10/2/0)	0.20	±	0.03 ^b^_2_	0.22	±	0.08 ^b^_2_	0.57	±	0.06 ^a^
Coating 29 (10/2/0.5)	0.21	±	0.15 ^b^_2_	0.41	±	0.01 ^b^_1_	0.70	±	0.09 ^a^
Coating 31 (10/2.5/0)	0.21	±	0.06 ^b^_2_	0.17	±	0.01 ^b^_2_	0.83	±	0.11 ^a^
	**Antioxidant activity**								
	**Day 0**			**Day 30**			**Day 60**		
	Mean		SD *	Mean		SD	Mean		SD *
Control	27.35	±	2.24 ^a^	22.49	±	2.65 ^a^	4.23	±	0.06 ^b^_2_
Coating 28 (10/2/0)	22.78	±	1.53 ^b^	23.94	±	2.31 ^b^	34.03	±	5.40 ^a^_1_
Coating 29 (10/2/0.5)	28.36	±	1.44	21.47	±	4.78	27.31	±	0.28 _1_
Coating 31 (10/2.5/0)	26.45	±	1.66	17.80	±	0.72	27.58	±	3.54 _1_

* SD: standard deviation; ^a,b^ Values with different superscripts show significant differences (*p* < 0.05) between sampling days within the same batch. _1,2,3_ Values with different subscripts show significant differences (*p* < 0.05) according to the Tukey test between different batches on a sampling day.

**Table 8:** Results of pH, citric acid (g/L), and malic acid (g/L) of the figs analyzed during the shelf life study.

Batches	pH								
	Day 0			Day 30			Day 60		
	Mean		SD *	Mean		SD	Mean		SD *
Control	6.24	±	0.00 ^a^_1_	6.30	±	0.01 ^a^	5.01	±	0.01 ^b^_2_
Coating 28 (10/2/0)	5.98	±	0.02 ^b^_2_	6.28	±	0.03 ^a^	5.18	±	0.04 ^c^_1_
Coating 29 (10/2/0.5)	6.07	±	0.05 ^b^_2_	6.25	±	0.01 ^a^	5.21	±	0.01 ^c^_1_
Coating 31 (10/2.5/0)	6.14	±	0.02 ^a^_2_	6.21	±	0.02 ^a^	5.00	±	0.02 ^b^_2_
	**Citric acid**								
	**Day 0**			**Day 30**			**Day 60**		
	Mean		SD *	Mean		SD	Mean		SD *
Control	0.76	±	0.06 ^b^	2.02	±	0.23 ^a^_1_	0.84	±	0.14 ^b^
Coating 28 (10/2/0)	1.14	±	0.01 ^a.b^	1.43	±	0.16 ^a^_2_	0.78	±	0.04 ^b^
Coating 29 (10/2/0.5)	1.03	±	0.01 ^b^	2.16	±	0.00 ^a^_1_	0.70	±	0.09 ^c^
Coating 31 (10/2.5/0)	0.99	±	0.06 ^b.c^	1.96	±	0.32 ^a^_1_	0.86	±	0.03 ^c^
	**Malic acid**								
	**Day 0**			**Day 30**			**Day 60**		
	Mean		SD *	Mean		SD	Mean		SD *
Control	0.73	±	0.05 ^b^	2.12	±	0.23 ^a^_1_	0.88	±	0.16 ^b^
Coating 28 (10/2/0)	1.09	±	0.01 ^a.b^	1.52	±	0.18 ^a^_2_	0.81	±	0.03 ^b^
Coating 29 (10/2/0.5)	0.99	±	0.01 ^b^	2.26	±	0.00 ^a^_1_	0.73	±	0.10 ^b^
Coating 31 (10/2.5/0)	0.95	±	0.05 ^b^	2.05	±	0.34 ^a^_1_	0.95	±	0.04 ^b^

* SD: standard deviation; ^a,b,c^ Values with different superscripts show significant differences (*p* < 0.05) between sampling days within the same batch. _1,2_ Values with different subscripts show significant differences (*p* < 0.05) according to the Tukey test between different batches on a sampling day.

**Table 9:** Results of soluble solids (°Brix), glucose (g/L), and fructose (g/L) of the figs during the shelf-life study.

Batches	Soluble Solids								
	Day 0			Day 30			Day 60		
	Mean		SD *	Mean		SD	Mean		SD *
Control	79.40	±	0.50 ^c^_1_	85.30	±	0.70 ^a,b^	89.03	±	0.58 ^a^
Coating 28 (10/2/0)	73.47	±	1.55 ^b^_2_	84.00	±	0.75 ^a^	87.20	±	0.26 ^a^
Coating 29 (10/2/0.5)	73.53	±	1.80 ^b^_2_	83.30	±	0.52 ^a^	85.87	±	0.25 ^a^
Coating 31 (10/2.5/0)	74.53	±	2.03 ^b^_2_	83.07	±	1.91 ^a^	85.37	±	0.45 ^a^
	**Glucose**								
	**Day 0**			**Day 30**			**Day 60**		
	Mean		SD *	Mean		SD	Mean		SD *
Control	109.44	±	9.52 ^a^	20.21	±	4.08 ^b^	19.37	±	0.85 ^b^
Coating 28 (10/2/0)	72.91	±	5.64 ^a^	20.11	±	1.16 ^b^	16.40	±	0.53 ^c^
Coating 29 (10/2/0.5)	95.62	±	1.56 ^a^	12.35	±	4.73 ^b^	20.83	±	0.03 ^c^
Coating 31 (10/2.5/0)	52.91	±	6.96 ^a^	22.74	±	6.80 ^b^	17.65	±	1.45 ^b^
	**Fructose**								
	**Day 0**			**Day 30**			**Day 60**		
	Mean		SD *	Mean		SD	Mean		SD *
Control	102.47	±	12.29 ^a^_1_	19.56	±	3.05 ^b^	19.21	±	1.80 ^b^
Coating 28 (10/2/0)	64.47	±	3.58 ^a^_1_	18.52	±	1.39 ^b^	14.95	±	0.97 ^b^
Coating 29 (10/2/0.5)	82.32	±	5.11 ^a^_1_	20.55	±	1.94 ^b^	18.95	±	0.37 ^b^
Coating 31 (10/2.5/0)	47.04	±	5.99 ^a^_2_	20.71	±	6.46 ^b^	17.33	±	2.19 ^b^

* SD: standard deviation; ^a,b,c^ Values with different superscripts show significant differences (*p* < 0.05) between sampling days within the same batch. _1,2_ Values with different subscripts show significant differences (*p* < 0.05) according to the Tukey test between different batches on a sampling day.

## Data Availability

Data are contained within the article.
